# RETRACTION: Effects of Tourniquet on Surgical Site Wound Infection and Pain After Total Knee Arthroplasty: A Meta‐Analysis

**DOI:** 10.1111/iwj.70289

**Published:** 2025-03-03

## Abstract

**RETRACTION:** LinZ. , ChenT. , ChenG. , PanW. , and XuW. , “Effects of Tourniquet on Surgical Site Wound Infection and Pain after Total Knee Arthroplasty: A Meta‐Analysis,” International Wound Journal 21, no. 2 (2024): e14414, 10.1111/iwj.14414.PMC1082462237779328

The above article, published online on 01 October 2023, in Wiley Online Library (http://onlinelibrary.wiley.com/), has been retracted by agreement between the journal Editor in Chief, Professor Keith Harding; and John Wiley & Sons Ltd. Following an investigation by the publisher, all parties have concluded that this article was accepted solely on the basis of a compromised peer review process. The editors have therefore decided to retract the article. The authors did not respond to our notice regarding the retraction.

**RETRACTION:** Z. Lin , T. Chen , G. Chen , W. Pan , and W. Xu , “Effects of Tourniquet on Surgical Site Wound Infection and Pain after Total Knee Arthroplasty: A Meta‐Analysis,” International Wound Journal 21, no. 2 (2024): e14414, 10.1111/iwj.14414.PMC1082462237779328

The above article, published online on 01 October 2023, in Wiley Online Library (http://onlinelibrary.wiley.com/), has been retracted by agreement between the journal Editor in Chief, Professor Keith Harding; and John Wiley & Sons Ltd. Following an investigation by the publisher, all parties have concluded that this article was accepted solely on the basis of a compromised peer review process. The editors have therefore decided to retract the article. The authors did not respond to our notice regarding the retraction.

